# Health problems of Iraqi police dogs referred to Baghdad Veterinary Hospital during 2015-2017

**DOI:** 10.14202/vetworld.2019.1046-1051

**Published:** 2019-07-16

**Authors:** Naqa Saleh Mahdi Tamimi, Abdulraheem Abduljalil Wali

**Affiliations:** 1 Department of Internal and Preventive Medicine, College of Veterinary Medicine, Wasit University, Iraq; 2 Department of Small Animal, Baghdad Veterinary Hospital, Baghdad, Iraq

**Keywords:** health problems, Iraq, police dogs

## Abstract

**Background and Aim:**

Police dogs in Iraq have been working mostly as explosive detectors since 2003. The health problems of these dogs are unique and have not been reported in literature. This investigation assessed the prevalence of health problems in Police dogs referred to Baghdad Veterinary Hospital during 2015-2017.

**Materials and Methods:**

A total of 1220 police dogs that were referred to Baghdad Veterinary Hospital in 2015-2017 were studied. The dogs were mostly German Shepherd dogs (GSDs) or Belgian Malinois (BM), with an average age of 4.6 years. The dogs’ health problems and some of their risk factors were studied.

**Results:**

Congestive heart failure (CHF), babesiosis, various malignancies, and intestinal parasites were the most commonly diagnosed health problems, followed by general aging, bronchopneumonia, otitis, nutritional deficiencies, and anemia. GSDs were more prone to CHF, while BM had more diagnoses of malignancies. Age was associated with both health conditions.

**Conclusion:**

The presence of health problems in these working dogs highlights the need for a stricter and more organized preventive schedule to keep the dogs healthy and efficient at old age.

## Introduction

For decades, dogs have been used for scent detection and are deployed daily worldwide to detect drugs, explosives, and firearms; as well as finding missing people and escaped fugitives [1,2].

The uses of working dogs are expanding in many parts of the world, especially in places where conventional security systems have failed. Iraq, especially after the invasion in 2003, is an example of those places in which no conventional military system has proved useful to detect firearms and explosives as effectively as dogs. As a result, thousands of dogs have been imported since then to meet these demands and have cost the Iraqi government millions of dollars. “Police dogs” is the term used to describe these dogs in Iraq; however, most of these dogs are military working dogs (MWDs), and they work as explosive detectors and sometimes patrol dogs. These dogs are distributed in various places in the country, such as army facilities, police checkpoints, airports, ministries, hotels, and other potential bombing targets.

Police dogs live unique lives and have special needs; thus, keeping them healthy and effective requires additional costs, effort, and expertise. Years after the massive deployment and use of police dogs in Iraq and the aging of these dogs, their health issues started to reveal themselves, and many of them are being sent to the veterinary hospitals for assessment and examination almost every day. Numerous studies regarding diseases of dogs have been published throughout the world [3-7]. For example, diarrhea and vomiting, otitis externa, pyoderma, and conjunctivitis were reportedly the most commonly diagnosed conditions in the dogs in three practices in the UK in 2007-2009 [8]. Furthermore, a telephone survey in the United States and Australia, observing 635 dogs, indicated that the most common health problems were musculoskeletal, dental disease, and gastrointestinal/hepatic problems [9]. Locally, a prevalence of canine diseases referred to private practice in 2015 and 2016 in Baghdad was described in a recent study, which reported that infectious (canine distemper and parvovirus infections) and gastrointestinal diseases were the most common health problems of these dogs [10]. The age, environment, and working schedule of the police dogs make them very different from house pets, in addition to their important role in providing protection and security to the country. With increasing age, it is essential that their situation is studied. To the best of our knowledge, no studies have been published on police dogs in Iraq.

Therefore, the purpose of this investigation was to assess the prevalence of common health problems and their risk factors in Iraqi police dogs referred to Baghdad Veterinary Hospital during 2015-2017.

## Materials and Methods

### Ethical approval

All individual information of dogs and those regarding their health issues were obtained with the full approval of the Manager of the Veterinary Hospital without revealing their identities in any way.

### Study population

Paper health files of police dogs referred to Baghdad Veterinary Hospital, located in Adan Square, Baghdad, which is the specialized center for small animals in Baghdad, from January 1, 2015, to December 31, 2017, were retrieved from the hospital archives and recorded. Many of the institutions in which these dogs serve have their veterinary care centers that deal with minor or emergency health issues; therefore, the dogs referred to the hospital do not completely reflect all diseased dogs.

The following data were extracted from the dogs’ health files: Date, name, age, sex, and breed of dogs, in addition to the health condition of the animal. The referral institution was not required to keep the privacy of those institutions intact, and only the governance of the dog’s duty was recorded. All the referred dogs were included in the study, including puppies before recruitment.

### Health conditions

Referred police dogs underwent a physical examination in the presence of their handlers. Radiology (AMERICOMP, V125), ultra-sonography (SonoSite, VET 180PLUS), and laboratory tests were also used to help reach a diagnosis. Available laboratory tests included blood films for tick-borne diseases, such as babesiosis; Rose Bengal for brucellosis; culture and direct smears for fungi; and commercial diagnostic kits for canine distemper, parvovirus, and heartworm infections. Conventional X-ray was used to confirm congestive heart failure (CHF). Unfortunately, no confirmatory test was available for *Brucella canis* or *Babesia* in the hospital, and even the mentioned diagnostic techniques were unavailable at some points.

### Statistical analysis

The reported health problems were grouped, and the more commonly reported ones were analyzed using logistic regression models. Therefore, the possible presence of any association between these health problems and the dogs’ characteristics, in addition to their odds ratios, were studied. The analyzed dogs’ characteristics included their age, sex, and breed. The most referred police dogs were from Baghdad (1061 of 1220); therefore, the location of duty was not considered in the data analysis (SPSS software, version 20; SPSS Inc. Chicago, IL, USA).

## Results

The main characteristics of the 1220 police dogs participating in this study are shown in Table-1. The neutering status of dogs was not recorded in their files, but on asking, it was mentioned that these dogs were rarely neutered (Table-1). The minimum age of subjects in this study was 2 months, and the maximum age was 14 years, with 43 dogs aged 10 years and more. The mode and median for age were 7 and 4 years, respectively.

**Table 1:** Main characteristics of police dogs referred to Baghdad veterinary hospital in 2015-2017.

Characteristics	n (%)
Breed	
German shepherd	828 (67.9)
Belgian Malinois	314 (25.7)
Labrador retriever	25 (2)
Dutch Shepherd	7 (0.6)
Cocker spaniel	3 (0.2)
Not specified	41 (3.4)
Hound dog	1 (0.1)
Mix	1 (0.1)
Sex	
Male	778 (63.8)
Female	440 (36.1)
Not registered	2 (0.1)
Age (years) mean±SD 4.58±2.95	

SD=Standard deviation

Most dogs (1061) were referred from Baghdad Province, while 103 were referred from Wasit, which is near Baghdad, and the remaining 56 came from other provinces around the country. The prevalence of health conditions diagnosed in the dogs referred to Baghdad Veterinary Hospital from 2015 to 2017 is shown in Table-2.

**Table 2:** Detailed diagnosed health problems of referred police dogs to Baghdad veterinary hospital in 2015-2017.

Categories	Diagnosed health problems (number of dogs/%)	Total (%)
Cardiovascular system	Congestive heart failure (193/15.8); pulmonary edema (85/7); arrhythmia (37/3); pericarditis (3/0.25); ventricular tachycardia (2/0.16)	235**(19.3)
Digestive system	Intestinal parasites (93/7.6); periodontal problems (27/2.2); enteritis (28/2.3); gastroenteritis (12/1); gastritis (8/0.66); megacolon (9/0.74); gastric ulcer (4/0.33); intestinal obstruction (2/0.16); cholecystitis (2/0.16); cirrhosis (2/0.16); IBS (1/0.08); mucocele (1/0.08)	189 (15.5)
Infectious diseases	Babesiosis (108/8.9); brucellosis (33/2.7); canine distemper (15/1.2); canine parvovirus (6/0.5); neosporosis (6/0.5); coccidiosis (4/0.3); anaplasmosis (3/0.25); giardiasis (3/0.25); meningitis (1/0.1)	179 (14.7)
Poor management and aging*	Overworking and nutritional deficiency (36/3); anemia (32/2.6); aging (45/3.7)	113 (9.3)
Musculoskeletal system and trauma	Arthritis (22/1.8); hip dysplasia (21/1.7); wound (18/1.5); hematoma (15/1.3); mild trauma (13/1); osteoarthritis (9/0.73); fractures (4/0.33); dislocation (4/0.33); cranial cruciate ligament rupture (4/0.33); osteoporosis (1/0.08); hernia (1/0.08); bullet (1/0.08)	113 (9.27)
Skin, eye and ear	Dermatophytosis (27/2.2); dermatitis (3/0.24); abscess (5/0.41); surgery site infection (3/0.24); anal gland infection (7/0.57); blindness (8.065); KCS (5/0.41); cataract (1/0.08); *Thelazia callipaeda* (1/0.08); decreased vision (2/0.16); otitis (36/3); deafness (1/0.08); decreased hearing (1/0.08)	100 (8.2)
Respiratory system	Upper respiratory infection (6/0.5); bronchitis (26/2.1); bronchopneumonia (37/3.0); pulmonary emphysema (6/0.5); pleural effusion (2/0.2)	77 (6.3)
Other	malignancies (45/3.7); epilepsy (1/0.1); hypothyroidism (1/0.1)	46** (3.8)
Reproductive system	Pyometra (9/0.74); orchitis (8/0.65); endometritis (4/0.33); castration (3/0.25); vaginitis (4/0.33); ovarian cyst (3/0.25); unilateral cryptorchidism (2/0.16); benign prostatic hyperplasia (1/0.08); ovariohysterectomy (5/0.41)	39 (3.2)
Urinary system	Urinary tract infection (26/2.1); urolithiasis (8/0.65); renal failure (3/0.25)	37 (3.0)
Behavioral problems	Tail biting-OCD (8/0.65); aggression (1/0.08)	9 (0.73)

* General signs of aging such as reduced activity level with no specific diagnosed health condition

** Some dogs were reported with more than one health condition, so the overall rate will surpass the total

Results of the data analysis and the association between the more common health problems in the referred dogs (babesiosis, CHF, various malignancies, and intestinal parasites) and the dogs’ characteristics (age, sex, and breed) are shown in Table-3. Babesiosis and intestinal parasites were not associated with any of the dogs’ characteristics, while CHF was associated with sex and age, and various malignancies were associated with breed and age (Table-3).

**Table 3:** Significant associations between the most prevalent diagnoses and the dogs’ characteristics and their odds ratios.

Disease/parameters	Significant	Exp. B (odds ratio)
CHF		
Sex (M/F)	0.006	1.65
Age	0	1.28
Breed (GSD/BM)	0.003	1.87
Period (1^st^/2^nd^ six M)	>0.05	
Malignancies		
Sex (M/F)	>0.05	
Age	0	1.37
Breed (BM/GSD)	0.007	2.48
Period (1^st^/2^nd^ six M)	0.02	2.16
Intestinal parasites*	>0.05	
Babesiosis*	>0.05	

* All four dogs’ characteristics did not show any association. CHF=Congestive heart failure, BM=Belgian malinois, GSD=German Shepherd dogs

## Discussion

This investigation assessed the prevalence of diseases in 1220 police dogs referred to Baghdad Veterinary Hospital from 2015 to 2017. The average age of dogs participating in this study was 4.58±2.95 (SD) years, with 43 dogs aged 10 years and more (Table-1). This is higher than the 9 months mean reported in a local private practice earlier in Baghdad. The reason for this is the high reference of puppies in private practices for preventive medicine, while in police dogs the number of young puppies is limited to facilities in which a breeding program exists [10].

Another demographic study in the UK also reported that dogs aged 1 year or less were the highest referred proportion and that clinical visits decreased slightly with aging [11]. Most dogs (more than 90%) were German Shepherd dogs (GSDs) or Belgian Malinois (BM), the same breeds used in the United States as MWDs [12]. The third most common dog breed was the Labrador Retriever, with a far lower 2% of the study population, which served only as explosive detectors in public places.

Almost two-thirds of the participating dogs were male (Table-1) because they are generally known for being more aggressive than females and are preferred by the police. Moreover, the police are not interested in keeping female dogs along with males since they can easily distract each other or get into fights over females in heat (especially in our subjects, most of which were not neutered).

Cardiovascular diseases were the most common problems diagnosed in these dogs, with CHF being the most common condition (193 dogs). Martini *et al*. [6] pointed out that digestive problems were the most common, followed by dermatological problems and cardiovascular diseases. Conversely, dermatitis and otitis externa were reported to be the most common health problems in dogs [4]. A study published recently in South Korea suggested that heart diseases were one of the most common medical problems in dogs aged 10 years or more [7]. Pulmonary edema was reported in 85 CHF cases (Table-2). CHF in the present study was associated with sex, age, and breed (Table-3). Males were 60% more prone to symptoms of CHF than females. GSDs were 87% more likely to be diagnosed, and for each additional year of age, dogs were 28% more likely to be diagnosed with CHF. Males have previously been reported to have a lower threshold for acquired vulvar heart disease, and they seem to show the signs of CHF at a younger age than females [13]. Our results are also consistent with the finding that heart-related diseases are more common among old dogs [14]; however, this condition’s rates still seem high, and the authors suggest that the nutritional, environmental, or even genetic factors may have played a role in this finding.

Furthermore, Gough and Thomas [15] support our finding that German shepherds are predisposed to aortic stenosis and mitral and tricuspid insufficiencies that can eventually lead to CHF. Dogs with advanced stages of CHF and pulmonary edema are barely able to walk and therefore cannot work, and they are eligible candidates for retirement; otherwise, their welfare might be at stake. In our population, these dogs were usually given temporary rests. No official age is designated for police dogs’ consideration for retirement in Iraq.

Infectious diseases are usually controlled by vaccination and other biosecurity measures. A tick-borne disease such as babesiosis rated as high as 8.85% in this study, with no association with any of the dogs’ characteristics (Tables-2 and 3). However, not all animals showed clinical signs at the time of diagnosis. Variable rates of babesiosis have been reported worldwide, from 0.1% in Turkey to 87.8% in Lithuania [16,17]. The fact that *Babesia* species are transported transovarially through ticks implies that the infection may remain endemic in areas for a long time [18].

Furthermore, the Rose Bengal test proved positive in 33 dogs, some of which showed clinical signs of brucellosis. This finding highlights the risks of zoonotic diseases to handlers of police dogs. Furthermore, canine distemper and parvovirus infection were reported in puppies of facilities with the breeding system. A more secure hygienic environment and a proper vaccination system can lower the rates of infectious diseases in these dogs.

Intestinal parasites were recorded in 93 of 1220 dogs (7.6%) in this study (Table-2), and none of the dogs’ characteristics were associated with the diagnosis (Table-3). This percentage is very similar to what was reported in police dogs of Egypt compared to the high rate in house dogs in the same study [19]. It appears that despite biosecurity measures, regular deworming schedules, and the use of high-quality dry food for police dogs, various intestinal parasites are an ongoing problem. Other inflammatory problems of the gastrointestinal tract, such as gastritis and gastroenteritis, are reported less commonly in our study (Table-2). This is in contrast with the results of another study, which suggested that problems of the digestive system had been the most common, representing a high proportion of deaths (14.5%) of dogs aged younger than 3 years [20]. This variation can be due to a lack of reports of digestive problems such as gastric dilatation and volvulus in our study. This condition is probably underdiagnosed in our population since these dogs usually cannot get emergency X-rays due to the working hours of the hospital and the paperwork; they need to be referred to in the first place. In addition, the older age of the dogs studied herein could also be another reason for this difference or the fact that fatal infectious diseases of puppies, such as canine distemper or parvovirus infection, were counted in the infectious category. Overworking and nutritional deficiency were reported in 36 dogs, and anemia was reported in 32 dogs (Table-2). Police dogs are practically endurance dogs that need a very special diet. For example, the protein in their diet should be animal based, palatable, and between 30% and 40% of the diet, among other dietary recommendations [21]. Not all facilities seem to be able to keep up with the high standards of working dogs’ diet. Therefore, with the heavy workload that these animals have, in addition to some intestinal parasites, they can easily be prone to nutritional deficiency and anemia. Moreover, at least 45 dogs’ problems were related to their old age, and they seemed to have a low performance with their tasks.

It has been suggested that almost one-fourth of dogs referred to veterinary practices are diagnosed with musculoskeletal problems, with hip dysplasia being the most frequently diagnosed orthopedic disease [22]. Musculoskeletal problems comprised 6.4% of the health conditions in dogs referred to South Korea’s clinical facilities in 2016 [7]. Many of the dogs studied herein (114) were diagnosed with musculoskeletal problems, with 21 of them suffering from hip dysplasia (Table-2). This debilitating condition must be ruled out in newly recruited dogs since most dogs are either GSDs or BM, which are predisposed to this condition [15]. With the use of specific radiographic methods and examination, early detection of this developmental condition can be possible even before the age of one [23]. Arthritis and osteoarthritis (OA) were also reported (2.5%) in the participating dogs (Table-2). A study on dogs examined the effects of weight loss in dogs with hip dysplasia: A reduction of body weight in dogs was associated with significantly fewer lameness scores [24]. Today, exercise and activity management should be an essential aspect of therapy of animals with OA [21]. Injuries in police dogs on duty are not uncommon. In this study, mild trauma, fractures, dislocations, cranial cruciate ligament ruptures, and gunshots were reported in 13, 4, 4, 4, and 1 dog, respectively (Table-2). These injuries cannot be prevented in police dogs but can be treated accordingly. Veterinary staff is not usually specifically trained to manage severe cases of canine trauma, and most facilities do not have diagnostic imaging or full laboratory equipment. Therefore, the integration of veterinary care with human medical facilities in combat zones is vital to providing necessary veterinary care to critically injured dogs [25].

Dermatophytosis was the most common dermatologic problem and was reported in 27 (2.5%) dogs in this study, and its rate seemed much lower than that reported in Egypt’s police dogs (27.9%). Conversely, skin problems were generally reported as the most common health problems in companion dogs in South Korea [7,26]. The reason for this could be the preventive measures that police dogs have in our study since dermatophytosis is a contagious disease. In addition, 36 (0.3%) dogs in our study suffered from otitis, which is a rate similar to the study mentioned above in Egypt, revealing a very distinctive rate compared to the South Korean dogs’ rate (10.4%, Table-2) [7,26]. Large dogs with straight ears in our study (GSD and BM) seem to be less prone to otitis. Blindness and deafness are also expected in very old dogs (Table-2), although such dogs cannot serve as police dogs anymore.

Respiratory problems affected 77 (6%) dogs, with bronchopneumonia and bronchitis being the most common diagnosis (Table-3). This rate was similar to that of Korean companion dogs (5.9%) and lower than that of Egyptian police dogs (10.5%) [7,26].

Other health issues that can be diagnosed in aged dogs are various types of malignancies. As expected, the results of the current study suggested a 37% increase with each year of the dogs’ age (Table-3). BM dogs in our study were reported to be 2.5 times more prone to malignancies than their GSD counterparts. The BM’ predisposition to cancers versus GSDs was also suggested by a study in the US conducted on GSDs and BM dogs MWDs that died in 1992 [12]. Diseases of the reproductive and urinary system each affected about 3% of dogs separately in our study (Table-3), with the Egyptian dogs having a similar rate for problems of the urinary system [26].

Behavioral problems, such as aggression, were reported in one dog, while obsessive tail biting was reported in eight dogs (Table-2). In addition to the genetic component of the disorder, obsessive-compul­sive disorder (OCD) can be the result of unbalanced activity, lack of attention, or environmental stimulation, anxiety, and other reasons [27-29]. These dogs were those that had seriously injured their tail and led to a partial tail amputation. Therefore, it is suspected that the actual number of police dogs suffering from earlier stages of OCD or other behavioral problems is higher. The quality of the relationship between the handler and his/her military dogs was proved to influence the efficiency and welfare of these dogs, and this factor could play a role in the welfare of Iraqi police dogs since Iraqi dog handlers are not always familiar with dogs when they start working with them [30].

## Conclusion

The presence of various physical and mental conditions in the participating dogs of this study highlights the fact that despite preventive measures, these dogs can still suffer from many health problems, especially with their heavy-duty schedules. Police dogs in Iraq need to be not only active and healthy but also work many hours during the day, especially when there is a shortage of dogs to cover all necessary shifts and locations. Old age and chronic diseases, such as CHF, arthritis/OA, and nutritional deficiencies, can strongly affect the efficiency and welfare of a working dog. However, the lack of confirmatory tests may have affected the results of our study in some cases. All in all, aging is neither preventable nor can it be cured; however, it can be managed humanely.

## Recommendations

It is recommended that the working schedule of police dogs be modified as dogs pass the age of 6 or 7 years and an organized, complete checkup, including a thorough physical examination, radiology, and blood work (hematology and biochemistry) be repeated at least every 6 months to monitor their health. It is highly suggested that confirmatory tests be conducted before reporting disease in a dog, especially for zoonotic conditions or those with higher rates such as brucellosis, CHF, and babesiosis. Considering a dog’s retirement is also suggested after the age of 8. Moreover, reports of some zoonotic diseases such as brucellosis and dermatophytosis in this study also demonstrate the need for a stricter diagnosis and prevention program to safeguard the handlers and other peoples who are in contact with police dogs.

## Authors’ Contributions

NSMT designed the study. NSMT and AAW both contributed to extracting the data. NSMT analyzed the data and drafted the manuscript. Both authors have read and approved the final manuscript.

## References

[1] Gazit I, Terkel J (2003). Explosives detection by sniffer dogs following strenuous physical activity. Appl. Anim. Behav. Sci.

[2] Bird R.C (1997). Examination of the training and reliability of the narcotics detection dog. Ky. Law J.

[3] Genchi C, Bowman D, Drake J (2014). Canine heartworm disease (*Dirofilaria immitis*) in Western Europe:Survey of veterinary awareness and perceptions. Parasit. Vectors.

[4] O'Neill D.G, Church D.B, McGreevy P.D, Thomson P.C, Brodbelt D.C (2014). Prevalence of disorders recorded in dogs attending primary-care veterinary practices in England. PLoS One.

[5] Hossain S.S, Kayesh M.E (2014). Common diseases of pet animals in Dhaka city and their zoonotic importance. Int. J. Nat. Soc. Sci.

[6] Martini M, Busetto R, Cassini R, Drigo M, Guglielmini C, Masiero I, Menandro M.L, Pasotto D, Fenati M (2016). A surveillance system of diseases of small companion animals in the Veneto region (Italy). Int. J. Infect. Dis.

[7] Kim E, Choe C, Yoo J.G, Oh S.I, Jung Y, Cho A, Kim S, Do Y.J (2018). Major medical causes by breed and life stage for dogs presented at veterinary clinics in the Republic of Korea:A survey of electronic medical records. Peer J.

[8] Brodbelt D, Middleton S, O'Neill D, Summers J, Church D (2011). Companion animal practice based disease surveillance in the UK. Epidemiol. SantéAnim.

[9] Freeman L.M, Abood S.K, Fascetti A.J, Fleeman L.M, Michel K.E, Laflamme D.P, Bauer C, Kemp B.L, Van Doren J.R, Willoughby K.N (2006). Disease prevalence among dogs and cats in the United States and Australia and proportions of dogs and cats that receive therapeutic diets or dietary supplements. J. Am. Vet. Med. Assoc.

[10] Tamimi N (2017). Prevalence of diseases in the canine referred to a private practice in Baghdad in 2015-2016. Kufa J. Vet. Med. Sci.

[11] Sa´nchez-Vizcaı´no F, Noble P.J.M, Jones P.H, Menacere T, Buchan I, Reynolds S, Dawson S, Gaskell R.M, Evertt S, Radford A.D (2017). Demographics of dogs, cats, and rabbits attending veterinary practices in Great Britain as recorded in their electronic health records. BMC Vet. Res.

[12] Michael R.P, Allen F.R, Dale G.D (2000). A study of the lifetime occurrence of neoplasia and breed differences in a cohort of German shepherd dogs and Belgian Malinois military working dogs that died in 1992. J. Vet. Intern. Med.

[13] Olsen L.H, Haggstrom J, Peterson H.D, Stephan J.E, Edward C.F (2010). Acquired vulvar heart disease. Textbook of Veterinary Internal Medicine.

[14] Hamlin R.L (1987). Managing Cardiologic Disorders in Geriatric Dogs. Proceedings Geriatric Medicine Symposium.

[15] Gough A, Thomas A (2004). Breed Predispositions to Disease in Dogs and Cats.

[16] Aktas M, Ozubek S, Altay K, Ipek N.D, Balkaya I, Utuk A.E, Kırbas A, Şimsek S, Dumanlı N (2015). Molecular detection of tick-borne rickettsial and protozoan pathogens in domestic dogs from Turkey. Parasit. Vectors.

[17] Paulauskas A, Radzijevskaja J, Karveliene B, Grigonis A, Aleksandraviciene A, Zamokas G, Babickaitė L, Sabūnas V, Petkevičius S (2014). Detection and molecular characterization of canine babesiosis causative agent *Babesia canis* in the naturally infected dog in Lithuania. Vet. Parasitol.

[18] Solano-Gallego L, Sainz Á, Roura X, Estrada-Peña A, Miró G (2016). A review of canine babesiosis:The European perspective. Parasit. Vectors.

[19] Ahmed W.M, Mousa W.M, Aboelhadid S.M, Tawfik M.M (2014). Prevalence of zoonotic and other gastrointestinal parasites in police and house dogs in Alexandria, Egypt. Vet. World.

[20] O'Neill D.G, Church D.B, McGreevy P.D, Thomson P.C, Brodbelt D.C (2013). Longevity and mortality of owned dogs in England. Vet. J.

[21] Case L.P, Daristotle L, Hayek M.G, Raasch M.F (2011). Canine and Feline Nutrition:A Resource for Companion Animal Professionals.

[22] Johnson J.A, Austin C, Breuer G.J (1994). Incidence of canine appendicular musculoskeletal disorders in 16 veterinary teaching hospitals from 1980-1989. J. Vet. Comp. Orthop. Traumatol.

[23] Ginja M.M, Gonzalo-Orden J.M, Melo-Pinto P, Bulas-Cruz J, Orden M.A, San Roman F, Llorens-Pena M.P, Ferreira A.J (2008). Early hip laxity examination in predicting moderate and severe hip dysplasia in Estrela mountain dog. J. Small Anim. Pract.

[24] Impellizeri J.A, Tetrick M.A, Muir P (2000). Effect of weight reduction on clinical signs of lameness in dogs with hip osteoarthritis. J. Am. Vet. Med. Assoc.

[25] Baker J.L, Truesdale C.A, Schlanser J.R (2009). Overview of combat trauma in military working dogs in Iraq and Afghanistan. J. Spec. Oper. Med.

[26] Rakha G.M.H, Abdl-Haleem M.M, Farghali H.A.M, Abdel-Saeed H (2015). Prevalence of common canine digestive problems compared with other health problems at teaching veterinary hospital, faculty of veterinary medicine, Cairo University, Egypt. Vet. World.

[27] Overall K.L (2003). Medical differentials with potential behavioral manifestations. Vet. Clin. North Am. Small Anim. Pract.

[28] Fox M.W (1972). Understanding your Dog.

[29] Thompson W.R, Melazack R, Scott T.H (1956). Whirling behavior in dogs as related to early experience. Science.

[30] Lefebvre D, Diederich C, Delcourt M, Giffroy J.M (2007). The quality of the relation between handler and military dogs influences efficiency and welfare of dog. Appl. Anim. Behav. Sci.

